# The Safety and Efficacy of Vascular-Targeted Photodynamic Therapy in Low-Risk Prostate Cancer

**DOI:** 10.3390/cancers17040661

**Published:** 2025-02-16

**Authors:** Pietro Saldutto, Fernando Cavacece, Roberto La Rocca, Ernesto Di Mauro, Vittore Verratti, Giuseppe Massimo Sangiorgi, Walter Vena, Gianluigi Patelli, Fabrizio Iacono, Francesco Di Bello, Luigi Napolitano, Vincenzo Maria Altieri

**Affiliations:** 1 Department of Urology, Humanitas Gavazzeni, 24125 Bergamo, Italy; pietro.saldutto@gavazzeni.it (P.S.); fernando.cavacece@aslfrosinone.it (F.C.); 2 Urology Unit, Department of Neurosciences, Reproductive Sciences and Odontostomatology, University of Naples “Federico II”, 80138 Naples, Italy; roberto.larocca@unina.it (R.L.R.); ernesto.dimauro@unina.it (E.D.M.); fran.dibello12@gmail.com (F.D.B.); 3 Department of Psychological, Health and Territorial Sciences, University “G. d’Annunzio” Chieti-Pescara, 66100 Chieti, Italy; vittore.verratti@unich.it; 4 Department of Biomedicine and Prevention, University of Rome Tor Vergata, 00133 Rome, Italy; gsangiorgi@gmail.com; 5 Endocrinology, Humanitas Gavazzeni, 24125 Bergamo, Italy; walter.vena@gavazzeni.it; 6 Department of Biomedical Sciences, Humanitas University, 20090 Milan, Italy; 7 Radiology Department, Casa di Cura San Francesco, 24128 Bergamo, Italy; gianluigi.patelli@cdcsanfrancesco.it; 8 Department of Medicine and Health Sciences “V. Tiberio”, University of Molise, 86100 Campobasso, Italy; info@fabrizioiacono.it (F.I.); vincenzo.altieri@unimol.it (V.M.A.); 9 Unit of Urology, Azienda Sanitaria Locale (ASL) Salerno DS-66, 84125 Salerno, Italy

**Keywords:** prostate cancer, urological cancer, vascular-targeted phototherapy, TOOKAD^®^, padeliporfin

## Abstract

Background: Prostate cancer (PCa) is one of the most prevalent cancers in the world. Standard methods of screening and diagnosis for prostate cancer have been effective but can result in overtreatment of indolent prostate cancer, leading to increased morbidity. Multiparametric magnetic resonance imaging (MRI) and fusion biopsy are effective tools to achieve better diagnostic accuracy. A combination of multiparametric MRI and photodynamic therapy can be used as an alternative to active surveillance in low-risk prostate cancer to better detect disease progression while avoiding overtreatment. Methods: We conducted a retrospective multicenter study on 13 patients with low-risk prostate cancer who underwent vascular-targeted photodynamic therapy. The patients were evaluated for up to 15 months after the procedure using biochemical parameters like serum Prostate Specific Antigen (PSA), digital rectal examination, multiparametric MRI, and functional parameters like the International Prostate Symptom Score (IPSS), the 15-question International Index of Erectile Function questionnaire (IIEF-5), quality of life score (QoL), the International Consultation on Incontinence Questionnaire-Short Form (ICIQ-SF), and a uroflowmetry examination. Results: The patients did not experience any significant complications during or after the treatment. A decrease in serum PSA and prostate volume was observed from 7.38 ng/mL to 3.8 ng/ml with functional improvement evidenced by a decrease in the IPSS (from 15.4 to 11), QoL (from 3.15 to 2), and the IIEF-5 (from 17.23 to 16) score, and an improvement in uroflowmetry. Conclusion: Vascular-targeted photodynamic therapy is a safe and effective alternative to active surveillance in patients with low-risk prostate cancer.

## Simple Summary

Nowadays, several techniques have been developed for the management of prostate cancer. Focal therapy represents a choice to limit complications as well as sparing the neurovascular bundles, sphincter, and urethra. The aim of this paper is to evaluate the safety and efficacy of vascular-targeted photodynamic therapy in low-risk PCa. Furthermore, studies are necessary to investigate the possibility to include VTP as part of a multimodal approach to provide a synergic therapeutic effect in PCa management.

## 1. Introduction

Prostate cancer (PCa) is the third-most frequent malignancy after female breast and lung cancer [1]. In 2020, 1,414,259 new cases of prostate cancer were registered worldwide, representing 14.1% of all cancers in men [2]. A striking difference in incidence rates exists in different regions of the world, with the highest age-standardized incidence rates in Ireland (83.4/100,000) and the lowest in South Central Asia (6.3/100,000) [3]. For localized PCa, curative treatments as well as total prostatectomy, external radiotherapy, or brachytherapy are considered the gold standard [4,5,6]. Although the efficacy of these treatments has been demonstrated in high- and intermediate-risk PCa, their value in overall survival or specific survival in the treatment of low-risk cancers is controversial [7,8,9]. An alternative to these curative treatments is active surveillance (AS) but only low-risk PCa is amenable to this mode of management [10]. Recent decades have seen a trend towards earlier diagnosis of PCa because of greater public and professional awareness, leading to the adoption of both formal and informal screening strategies [11,12]. Therefore, PCa is diagnosed while still in the early stages with smaller tumors that occupy only 5–10% of the prostate volume, with a greater propensity for unifocal or unilateral disease [13,14,15]. Nowadays, in these cases, focal therapy has been found to be an effective alternative [16]. The aim of focal therapy is to ablate tumors selectively whilst limiting complications by sparing the neurovascular bundles, sphincter, and urethra [17,18,19]. Most focal therapies have been developed, and they utilize several ablative technologies as well as cryotherapy, High-Intensity Focused Ultrasound (HIFU), photodynamic therapy, electroporation, and focal radiotherapy by brachytherapy or CyberKnife^®^ Robotic Radiosurgery System technology (Accuray Inc., Sunnyvale, CA, USA) [20]. Vascular-targeted photodynamic therapy (VTP) represents one of the new technologies developed in PCa management. This is a minimally invasive technique used for patients with unilateral very-low-risk and low-risk PCa [21]. VTP is characterized by intravenous injection of a photosensitizer locally activated by laser fibers with a specific wavelength of light [22]. This activation produced free radicals that cause a local vascular injury followed by coagulative tissue necrosis [23]. According to previously published studies, it represents a promising treatment option in unilateral low-risk PCa, although a rigorous surveillance strategy is required [24]. In this multicenter retrospective study, we aim to evaluate the safety and efficacy of vascular-targeted photodynamic therapy in low-risk PCa.

## 2. Materials and Methods

We conducted a retrospective multicenter study between May 2019 and September 2020 in two centers in Italy (Department of Urology, Humanitas Gradenigo hospital and Department of Urology, University of Naples Federico II). We enrolled 13 patients with low-risk PCa treated with VTP. Ethics approval was obtained, and all patients consented to participating in the study. Data were retrieved from an Italian registry developed to gather information about this new technique. Data were collected about comorbidities, previous prostatic surgery, and family history of PCa. Each patient underwent a physical examination, a preliminary serum PSA (ng/mL) assessment, and a fusion biopsy based on mpMRI (PIRADS ≥ 3) results. Patients were also evaluated using IPSS, QoL score [25], IIEF-15) [26], ICIQ-SF [27], and a uroflowmetry examination. We assessed that each patient started follow-up with an mpMRI at 7 days, and at 6 and 12 months after VTP therapy. Patients were invited to undergo a repeat prostate biopsy in cases of increasing PSA in two consecutive exams, PIRADS ≥ 3 on mpMRI, or a suspicious DRE. The inclusion criteria were a life expectancy ≥ 10 years [28], a clinical stage of T1c or T2a, a Gleason Score ≤ 6 based on high-resolution biopsy strategies, PSA ≤ 10 ng/mL, three positive cores with a maximum cancer core length of 5 mm in any one core, or 1–2 positive cancer cores with ≥50% cancer involvement in any one core, or a PSA density ≥ 0.15 ng/mL/cm^3^ and volume of the prostate between 25 mL and 70 mL. Patients were excluded if their expected life expectancy was ≤10 years, if they had medical conditions that preclude the use of general anesthesia or MRI (e.g., cardiac pacemaker, bilateral hip replacement), any risk factor for VTP therapy, history of surgery for benign prostatic hypertrophy, hormonal manipulation, androgen deprivation, urethral stricture, or urinary retention within 6 months of the study.

### 2.1. Padeliporfin VTP Procedure

The VTP procedure was performed under general anesthesia. Patients received a single intravenous administration of Tookad (WST11, STEBA Biotech, L 2613 Luxembourg) at a dose of 4 mg/kg, followed by local illumination of the targeted area using 753 nm laser light at a power of 150 mW/cm and light energy of 200 J/cm delivered through transperineal optical fibers positioned in the prostate under ultrasound guidance [29]. The number and position of the fibers were adapted to each patient to obtain zonal to sub-total destruction of the areas of interest. Hemiablation (treatment of only one lobe of the prostate) was performed in patients with unilateral disease. The total duration of the procedure was 2 h. Patients remained under medical surveillance in dim light for at least 12 h. A urinary catheter was left in situ until the next morning. They were discharged the day after the procedure and were advised to avoid direct exposure to sunlight for 48 h. An alpha lytic was also recommended for at least one month.
**Padeliporfin VTP Procedure****Median (Range)**Operative time, median (range), minutes101.6 (70–135)Number of fibers, median (range)13 (7–20)

### 2.2. Oncological Outcome

The oncological outcome was assessed using serum PSA levels and prostate biopsy. Patients were followed up with a PSA every 3 months for 2 years and then every 6 months. A DRE was performed routinely. Each patient underwent an mpMRI at 7 days after treatment to evaluate the effect of the treatment and at 6 and 12 months to assess the treated and the untreated areas. If any patient had an increased PSA at 2 subsequent assessments or a suspicious DRE, a new biopsy was advised. In the absence of this, a prostate biopsy was recommended at 2, 4, and 7 years after VTP therapy. Progression was defined as a shift from the low-risk to the intermediate or high-risk group (Gleason score ≥ 6, PSA ≥ 10 ng/mL, T ≥ 2b). In cases of histological progression evidenced by prostate biopsy, radical treatment would be discussed.

## 3. Results

Patient characteristics are detailed in Table 1. All patients in the study had low-risk PCa with a median age of 63.4 years (range 50–74). PCa patients exhibited a median prostate volume of 53.53 (range 25–70), and the majority (*n* = 7) harbored a PIRADS 3 at mpMRI. The median number of positive cores was 1.4 (range 1–2), with a mean total cancer nucleus length of 2.8 mm (range 0.5–5.1).

Median follow-up was for 11.7 months (range 6–15). Eight patients (61.5%) were followed up for 12 months. Out of these eight patients, four were excluded by the follow-up due to a disease progression, and the remaining four (30.8%) continued the follow-up until the 15th month. Seven (53.8%) patients reported a total of 18 adverse effects within 30 days of the procedure. Most of them were mild to moderate, short-lived, and occurred soon after surgery. There was only one instance of decreased libido that occurred 3 months after the procedure. The most common adverse effects observed were hematuria and perineal pain in five patients (38.4%). The other adverse effects were transient dysuria, micturition urgency, hematospermia, glans erythema, and urinary retention. Urinary retention resolved within three days of catheterization (Table 2). New onset of urinary incontinence was not observed before or after the procedure. One patient had pre-existing urge incontinence that remained stable after VTP therapy.

**Figure 1:**
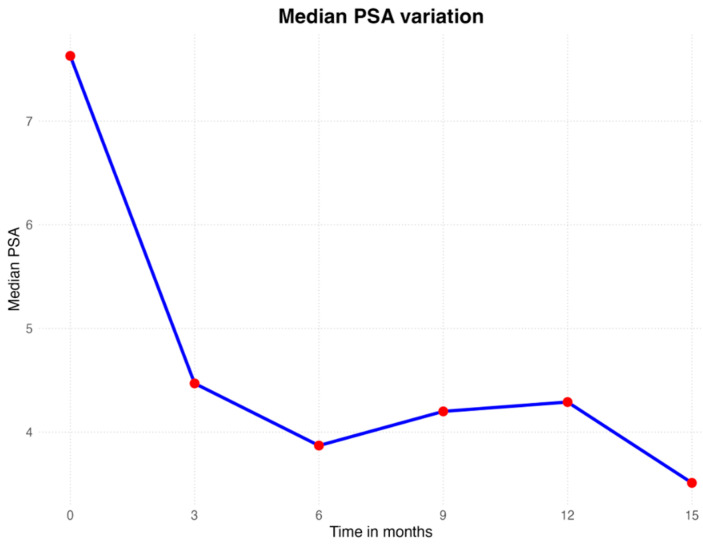
Median PSA level at 0, 3, 6, 9, 12, and 15 months.

**Figure 2:**
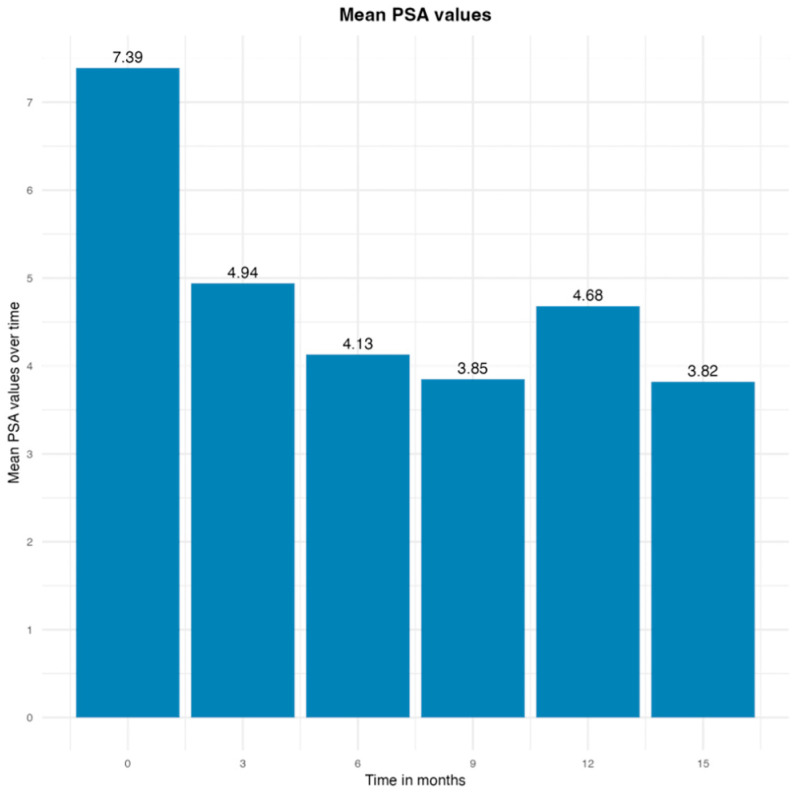
Average PSA level from baseline to 3, 6, 9, 12, and 15 months.

Median PSA range was 7.38 ng/mL at baseline, 4.93 ng/mL at three months, 4.13 ng/mL at six months, 3.56 ng/mL at nine months, 4.68 ng/mL at twelve months, and 3.8 ng/mL at fifteen months. At three months post-VTP, 92.7% (12/13) of the patients had a PSA decrease of 2.92 ng/mL. At six months post-VTP, 92.7% (12/13) of the patients had a PSA decrease of 3.6 ng/mL. At nine months post-VTP, 92.7% (12/13) of the patients had a PSA decrease of 3.8 ng/mL. At 12 months post-VTP, 53.8% (7/8) of the patients had a PSA decrease of 3.9 ng/mL. At 15 months post-VTP, 75% (3/4) of the patients had a PSA decrease of 3.2 ng/mL. Only one patient (7.6%) had oscillatory values of PSA within fifteen months of follow-up. The PSA trends are shown in Figure 1 and Figure 2.

**Figure 3:**
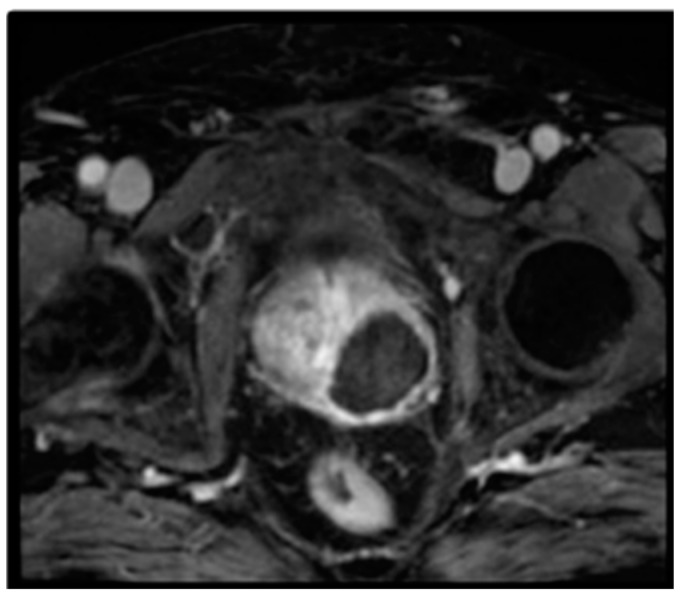
VTP focal hemiablation and mpMRI after 7 days.

All patients underwent mpMRI after the procedure at 7 days (Figure 3), and at 6 and 12 months. The prostate volume increased from baseline at 7 days after VTP: from a mean of 53.53 to 66.9 mL. There was a decrease in volume over time with a reduction to a median volume of 41.8 mL at 3 months and 40 mL at 6 months.

**Figure 4:**
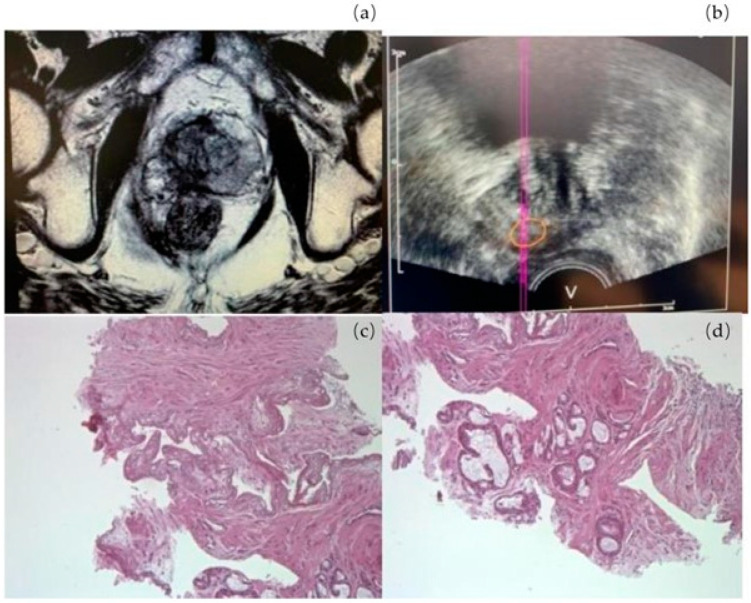
T2-weighted axial MRI images (PIRADS 4) (**a**); fusion biopsy (**b**); tumor area GS 6 (**c**); area of necrosis (**d**). The line and circle represent a machine function during biopsy.

In all patients, mpMRI performed 6 and 12 months after the procedure showed coagulative necrosis of the treated area. Only two patients underwent a prostate biopsy after one year, both due to a positive mpMRI. In one patient, the target region was PIRADS 4 in the non-treated lobe and, in the other subject (Figure 4), a PIRADS 3 area was adjacent to the treated zone. In the first patient, we performed a target biopsy in the ipsilateral and contralateral lobes. Two out of eight cores were positive in the treated lobe with Gleason score 6 (3 + 3) adenocarcinoma of the prostate. In this case, radical treatment and active surveillance were discussed, and the patient decided on a conservative option. In the second patient, we performed the fusion biopsy in the target zone and contralateral lobe, and the result was negative for cancer. The other patients did not undergo mpMRI due to the absence of clinical relapse suspicion. Prostate biopsy was not proposed at one year, by default, following the EMA/AIFA guidelines.

The mean IPSS decreased from 15.4 at baseline to 13.5 at three months, 13.38 at six months, 11.25 at twelve months, and 11 at fifteen months, indicating an overall reduction in urinary symptoms. At three months post-VTP, 69.7% (9/13) of the patients had an IPSS decrease of 1.1 points. We observed 23.0% (3/13) of patients with the same IPSS and one with an increase of 2 points compared to the baseline at three months. Six months post-VTP, 61.5% (8/13) of the patients had an IPSS decrease of 3.8 points. We observed 23.0% (3/13) of patients with an IPSS increase of 1.3, and two with the same scores compared to the baseline at six months. At 12 months post-VTP, 75% (6/8) of the patients had an IPSS decrease of 4 points. We have 12.25% (1/8) of patients with an increased IPSS of 2.3, and 12.25% (1/8) of patients with the same score compared to the baseline at twelve months. At 15 months post-VTP, 75% (3/4) of the patients had an IPSS decrease of 3.33 points. We have 25% (1/4) of patients with an increased IPSS of 2 compared to the baseline at fifteen months.

Mean uroflowmetry (Qmax) increased from 12.3 mL/s at baseline to 13.7 mL/s at three months, 13.0 mL/s at six months, 13.3 mL/s at twelve months, and 13 mL/s at fifteen months, indicating an overall reduction in urinary symptoms. We also saw 97.6% (12/13) of the patients had a Qmax increase of 0.95 mL/s after three months. We saw 7.6% (1/13) of patients with a Qmax decrease of 0.1 compared to the baseline at three months post-VTP. Six months post-VTP, 84.6% (11/13) of the patients had a Qmax increase of 0.96 mL/s. We saw 7.6% (1/13) of patients with an increased Qmax of 0.5 mL/s and 7.6% (1/13) of patients with the same score compared to the baseline at six months post-VTP. Twelve months post-VTP, 87.5% (7/8) of the patients had a Qmax increase of 1.3 mL/s. We saw 12.25% (1/8) of patients with a decrease of 0.2 compared to the baseline twelve months post-VTP. Fifteen months post-VTP, 50% (2/4) of the patients had a Qmax increase of 1.8 mL/s. We saw 25% (1/4) of patients with a decrease of 0.3 in score compared to the baseline and 25% (1/4) of patients with the same score compared to the baseline at fifteen months post-VTP. The quality-of-life questionnaires showed an improvement from the baseline. The mean score was 3.1 before surgery, 2.6 at three months, 2.31 at six months, 2.4 at twelve, and 2 at fifteen months. Three months post-VTP, 53.8% (7/13) of the patients had a quality of life decrease of 1. We saw 46.1% (6/13) of patients with the same QoL score compared to the baseline at three months. Six months post-VTP, 61.5% (8/13) of the patients had a QoL decrease of 1.3. We saw 38.4% (5/13) with the same score compared to the baseline at six months. Twelve months post-VTP, 62.5% (5/8) of the patients had a QoL decrease of 1.4. We have 23% (3/8) of patients with the same score compared to the baseline at twelve months. Fifteen months post-VTP, 75% (3/4) of the patients had a quality of life decrease of 1.5. We have 25% (1/4) of patients with the same QoL compared to the baseline at fifteen months. The mean IIEF-5 score was 17.2 at baseline, 16.1 at three months, 16.6 at six months, 16.5 ng/mL at twelve months, and 16 ng/mL at fifteen months. All patients retained ejaculatory function, and we also reported a case of a pregnancy in a young couple. Three months post-VTP, 84.6% (11/13) of the patients had an IIEF-5 decrease of 3.5. We saw 7.6% (1/13) of patients with the same IIEF-5 score and 7.6% (1/13) with an increase of 7 compared to the baseline at three months. Six months post-VTP, 38.4% (5/13) of the patients had an IIEF-5 decrease of 2.4. We saw 30.7% (4/13) of patients with the increase IIEF-5 of 1 and 30.7% (4/13) with the same score compared to the baseline at six months. Twelve months post-VTP, 50% (4/8) of the patients had an IIEF-5 decrease of 1.5. We have 37.5% (3/8) of patients with an increased IIEF-5 score of 1.6 and 12.5% (1/8) of patients with the same score compared to the baseline at twelve months. Fifteen months post-VTP, 50% (2/4) of the patients had an IIEF-5 increase of 1.5. We have 50% (2/4) of patients with the same IIEF-5 compared to the baseline at fifteen months. Our patients showed the same ICIQ-SF questionnaire score from baseline through follow-up.

## 4. Discussion

To the best of our knowledge, this is the first Italian multicentric study assessing the biochemical and functional outcomes of padeliporfin-targeted photodynamic therapy for low-risk PCa. Numerous studies have demonstrated the safety and feasibility of VTP therapy in low-risk PCa [30,31,32]. A study comparing disease progression between groups of patients who underwent VTP therapy or AS found that disease progression was seen at 24 months in 28% and 58% of patients in the VTP and AS groups, respectively. VTP therapy was well tolerated. The most common grade 3–4 adverse events were prostatitis, acute urinary retention, and erectile dysfunction, and the most common serious adverse event was urinary retention [33]. Thus, it was shown that partial-gland ablation influences the course of low-risk PCa, at least in the medium-term, by reducing the rate of detectable cancer in the treated lobes and by limiting the progression from a low-risk status to a higher one. However, it is important to note that performing VTP does not definitively preclude the possibility of a patient requiring a radical salvage treatment. In 2018, Gill et al. conducted a randomized trial comparing conversion to radical therapy between groups of patients who underwent VTP therapy and AS. The conversion rate was found to be lower in the ablation group than in the surveillance group, with 7%, 15%, and 24% conversion in the ablation group and 32%, 44%, and 53% conversion in the surveillance group at 2 years, 3 years, and 4 years, respectively [34]. Our results confirm the safety and the feasibility of VTP reported in the literature. We did not observe many adverse effects, and they were brief, mild to moderate, occurring soon after surgery, and relieved within a few days requiring only conservative management. In our study, we emphasize the importance of MRI for diagnosis as well as for defining the target area for biopsy. Our centers have extensive experience in fusion biopsy, making VTP feasible, effective, and safe. The limitation of our study is the lack of histopathological evidence during follow-up. Further studies that include long-term histopathological follow-up are required to better define oncological outcomes and support the data published in the literature. The biochemical indicator of treatment effectiveness we relied on in our study was a reduction in PSA value. This reduction can be explained by recovery from cancer (not confirmed by biopsies) and by the volume reduction of the prostate in the long term. We observed a sharp reduction in PSA values in the first trimester, and then a stabilization in the first year. In addition, no rectal examination revealed a recurrence in the prostatic loggia. Regarding functional outcomes, we observed an improvement in IPSS, Qmax, QoL, and IIEF-5 scores. All patients retained ejaculatory function, and one patient had a child. We can hence conclude that VTP does not seem to negatively affect the functional outcomes but rather improves them. No incontinence was observed during follow-up. This treatment is comparable with other types of focal therapies, as demonstrated in a review by Barret et al. in 2019 [35]. The main advantage of VTP seems to be the feasibility and the safety of radical treatment after VTP. Pierrard et al. conducted a retrospective study of 42 patients who underwent a salvage radical prostatectomy across 14 hospitals. The study showed similar outcomes as naïve patients who undergo the same surgery. Out of the salvage radical prostatectomies performed, 69% were considered easy. Only 12% of patients had postoperative complications which represent a comparable rate, confirming the safety of the salvage procedure [36]. Salvage prostatectomy post-VTP appears to have better functional outcomes than salvage prostatectomy performed post High-Intensity Focused Ultrasound (HIFU) ablation or radiotherapy [37]. We did not perform any salvage procedure. The only patient with cancer at biopsy was scheduled for AS considering the disease stability. VTP is an alternative treatment for specific subgroups of patients who are amenable to primary AS. This study presents some limitations: First, the retrospective nature of the study and the relatively small sample size of patients might result in biases related to the selection and treatment of patients.

## 5. Conclusions

Vascular-targeted photodynamic therapy is considered a safe treatment option with promising short-term oncological results and can represent an alternative to other focal treatments for localized PCa management or to active surveillance. It is a feasible and safe procedure, without major complications, and it can also improve urinary symptoms. Proper counseling about the risks and the side effects, along with rigorous surveillance, is necessary before and after the procedure. Patients also tend to find this to be a more favorable choice of treatment because of its low rate of complications such as incontinence and impotence, and because it can soothe the anxiety associated with the cancer and its progression. Despite this, further studies with a large sample size and longitudinal follow-up are required to confirm these data, and they are necessary to better define future clinical applicability not only in low-risk PCa. Furthermore, studies are necessary to investigate the possibility to include VTP as part of a multimodal approach to provide a synergic therapeutic effect in PCa management.

## Figures and Tables

**Table 1:** Clinical characteristics of patients enrolled in this study.

Baseline Characteristics	Median (Range) or n° (%)
Age, median (range), years	63.4 (50–77)
Weight, median (range), kg	74 (60–108)
Prostate volume, median (range), mL	53.53 (35–70)
PSA, median, (range), ng/ml	7.38 (2.7–9.8)
mpMRI (PIRADS)	3 (7) 4 (4) 5 (2)
Number of positive cores, median (range)	1.4 (1–2)
Median total cancer core length (range), mm	2.8 (0.5–5.1)
Gleason score	3 + 3 (13)
T-stage	T1c 7 T2a 6
IPSS, median, (range)	15.4 (8–30)
Uroflowmetry (Qmax), median, (range)	12.3 (9.5–18)
Quality of life (QoL) score, median, (range)	2.5 (1–4)
IIEF-5 score, median, (range)	17.2 (3–24)

**Table 2:** Adverse events reported by patients.

Adverse Event	n (%)
Any adverse event	18 (86%)
Hematuria	5 (27.7%)
Perineal pain	5 (27.7%)
Transient dysuria	3 (16.6%)
Micturition urgency	2 (11.1%)
Hematospermia	1 (5.5%)
Glans erythema	1 (5.5%)
Urinary retention	1 (5.5%)

## Data Availability

Data available on request.
